# Supplementary material for: Dysregulation of excitatory neural firing replicates physiological and functional changes in aging visual cortex
Source: PLoS Comput Biol. 2021 Jan 26;17(1):e1008620. doi: 10.1371/journal.pcbi.1008620 (PMC7864437; doi:10.1371/journal.pcbi.1008620)
Supplement: S1 Text — To support the main findings of our work we performed several additional analyses, including evaluating the orientation selectivity of hybrid networks created by mixing-and-matching young and old parameters sets, training the network with a critical learning period, demonstrating the excitatory neurons with similar receptive fields effectively inhibit one another (through inhibitory interneurons), and testing the history-dependence of training on the initial value of the excitatory target spike rate, pE(0). (PDF) [file pcbi.1008620.s001.pdf]

Supporting information for "Dysregulation of excitatory neural firing replicates physiological and functional changes in aging visual cortex"

Seth Talyansky[1,2 ¤], Braden A. W. Brinkman[2*]

1 Catlin Gabel School, Portland, Oregon, USA
2 Department of Neurobiology and Behavior, Stony Brook University, Stony Brook, New York, USA

¤ Current Address: Stanford University, Stanford, California, USA
* braden.brinkman@stonybrook.edu

Supporting figures

Several figures in the main text also have corresponding supplementary figures, briefly described here. In S1 Fig we show how the distributions of network parameters change over time, which displays histograms of the trained network parameters Q (input weights), W (lateral weights), and θ (firing thresholds) at several intermediate ages following network maturity (30 training loops). This expands on Fig 2 in the main text.

We test the robustness of our results by training the network on three different movies from the CatCam repository [40, 41] (`movie01.tar`, `movie07.tar`, and `movie16.tar`) and resetting all random number generation before each run. We find no appreciable qualitative differences across the results on the three image sets. See S2 Fig–S7 Fig.

Supplementary analyses

The figure numbering and references in this document continue from the main text.

To support the main findings of our work we performed several additional analyses, including evaluating the orientation selectivity of hybrid networks created by mixing-and-matching young and old parameters sets, training the network with a critical learning period, demonstrating the excitatory neurons with similar receptive fields effectively inhibit one another (through inhibitory interneurons), and testing the history-dependence of training on the initial value of the excitatory target spike rate, $p_E(0)$.

Evaluating network selectivity in hybrid networks of mixed young and old parameters

To support the numerical experiments discussed in "Numerical experiments to test contribution of different physiological parameters to declines in functional performance," we also perform a set of experiments in which we evaluate the orientation selectivity of several young-old hybrid networks that we create by mixing together different combinations of the young (age 30) and oldest (age 80) learned parameters; the learning rules are not active during these tests. As shown in Fig 8, the mean selectivity appears to be impacted most when input weights are old and thresholds are young (bars labeled "OYY" and "OOY", all movies). This is most likely because the input weights are smaller in magnitude in old age than in youth, and therefore the current inputs to each network version $\sum_k Q_{ik} X_k$ are comparatively smaller, whereas the young threshold values are adapted to correspondingly higher current inputs and therefore typically trend higher in order to achieve the target spike rates during development ($p_E(t_{\rm loop}) = 0.01$). Thus, typical current inputs drive the neurons to fire less. We see that replacing the young thresholds with the old-age thresholds improves the mean selectivity back to the pure old-age values (bars "OYO" and "OOO"). These results also

demonstrate that replacing the young lateral weights with old lateral weights does not appear to have a substantial impact on the mean selectivities, as simulations differing only in young/old lateral weights have very similar selectivities. Taken all together, this analysis supports the conclusions of our parameter freezing experiments (Fig 6) that the input weights are the most influential contributors to the decline in orientation selectivity, and any sharpening of selectivity that lateral weights might contribute is a second-order effect.

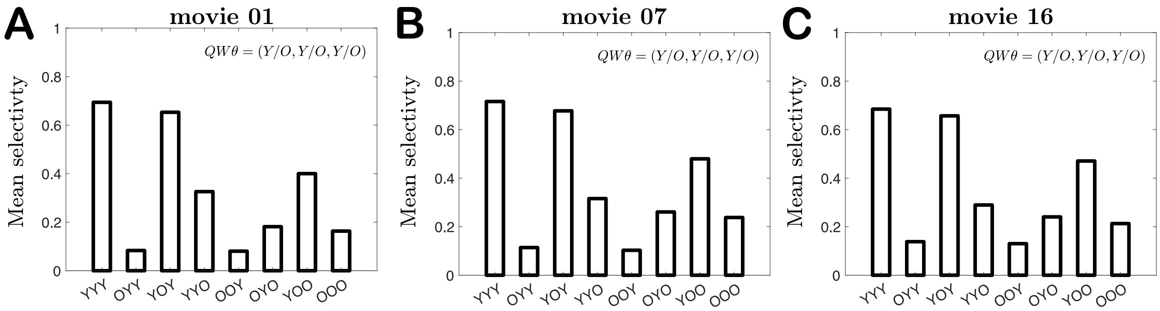

Fig 8. Mean selectivity in networks with swapped parameter sets: **A.** The mean orientation selectivity of neurons in network simulations in which we mix-and-match the parameter sets of young (30 loops) and old (80 loops) networks. Horizontal axis labels correspond to young (Y) or old (O) input weights (Q), lateral weights (W), and thresholds (θ). For example, the "OOY" result corresponds to a simulation using the old-age input weights and lateral weights but the young thresholds. This network was trained on `movie01` from the CatCam database [40, 41]. **B.** Same as panel **A** but for `movie07`. **C.** Same as panel **A** but for `movie16`.

Critical learning periods and time rescaling

The critical learning period hypothesis posits that there is a window early in development in which learning rates are high, which then taper off to lower levels of plasticity [72]. This is unlike our network model, in which the learning rates are the same throughout the training procedure (lifespan). To demonstrate that our training procedure does not preclude interpretation in terms of a critical learning period, we run a training case in which, after 30 loops, we reduce the global learning rate by a factor of 10 (thus reducing all network learning rates by the same factor) and extend the length of natural image stimulus presentation by a factor of 10, and then train the network for 50 more loops under these conditions. We find that average neural selectivity follows largely the same trend as in the case of normal aging (Fig 9). This suggests that networks with time-varying learning rates can be mapped to networks with constant learning rates by adjusting the length of training loops during these periods. The implication for our results is that we can interpret the development phase of our network as functionally equivalent to a critical learning period with higher rates and a shorter learning interval compared to the aging phase.

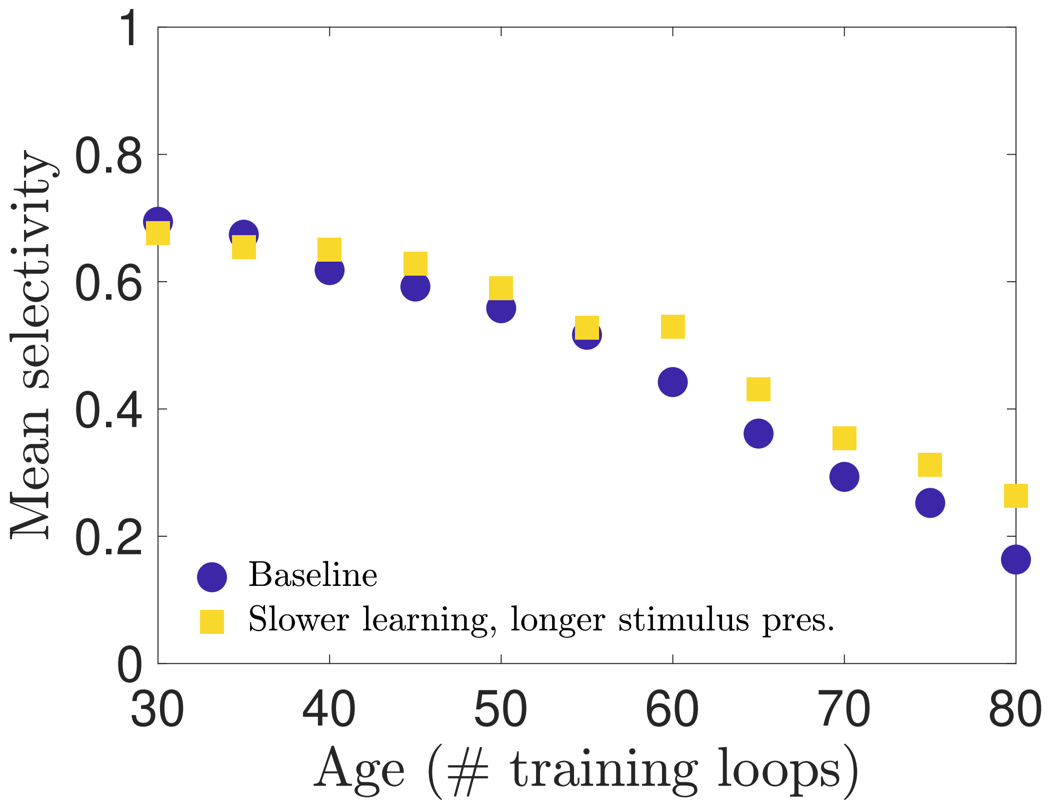

Fig 9. Testing selectivity in a network with a critical learning period: To show that one could in principle implement a critical learning period in the training phase of our network model, we show that if we train a new network in which learning rates are reduced by a factor of 10 and duration of training increased by 10 (representing the end of the critical learning period), the results are qualitatively similar. Thus, the training procedure employed in our model is expected to produce approximately the same results as a model with a critical period during which learning rates vary nonlinearly during training, so long as those changes are compensated by reciprocal changes in the duration of training. Results shown correspond to a network trained on `movie01` from the CatCam database [40, 41].

Excitatory neurons with similar receptive fields effectively mutually inhibit each other

To gauge the extent to which excitatory neurons with similar receptive fields inhibit one another, we take element (i, j) of the product of the lateral connection matrices W_{EI} and W_{IE} to approximate the net charge transfer capacity from pre-synaptic excitatory neuron i to post-synaptic excitatory neuron j, by way of all disynaptic pathways through inhibitory neurons. (Recall that in E-I Net there are no direct E-to-E connections; i.e., $W_{EE} = 0$). The greater the magnitude of $(W_{EI}W_{IE})_{ij}$, the greater the inhibition of excitatory neuron i by excitatory neuron j. As in Fig 6B of [39], we plot the RF overlap, computed as the cosine similarity of the vectorized input weight matrices, against the elements of $|W_{EI}W_{IE}|$ for all ordered pairs of excitatory neurons. In Fig 10 we see, similar to [39], that a larger overlap is generally associated with a larger in magnitude $W_{EI}W_{IE}$ value and thus, per our interpretation, stronger inhibition of one excitatory neuron in the pair by the other.

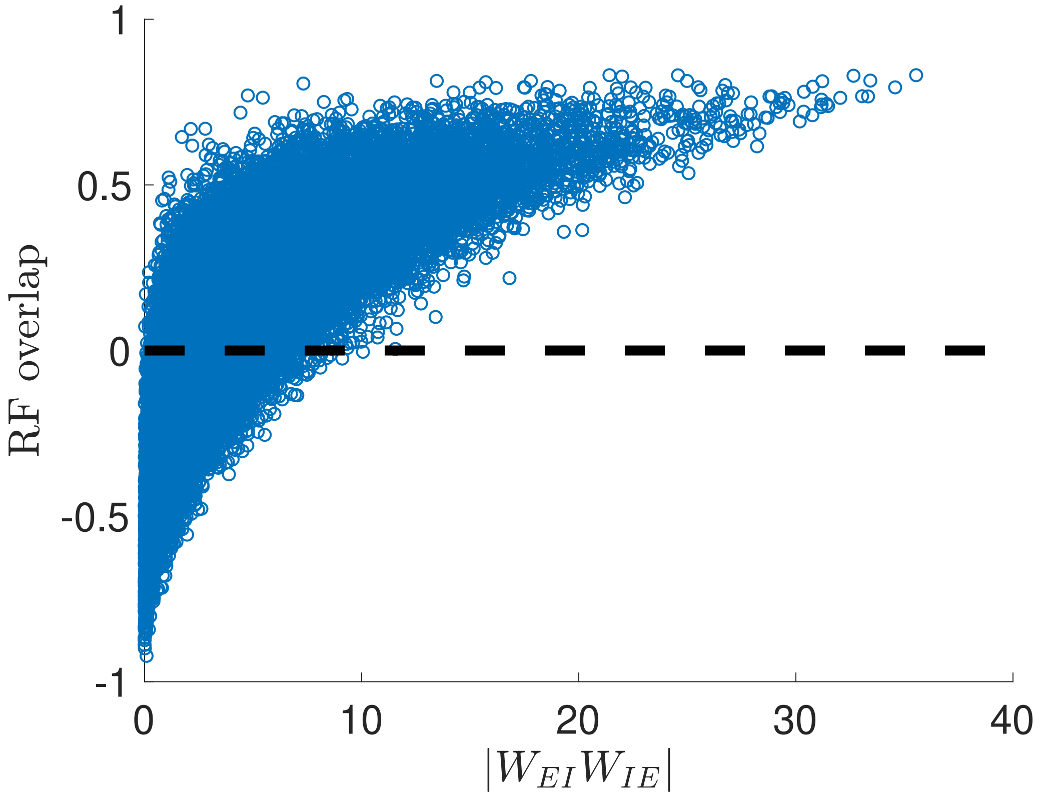

Fig 10. Excitatory neurons with similar receptive fields (RFs) effectively inhibit one another: As a proxy for the total amount of charge excitatory neurons transmit between each other, we use the elements of the matrix $W_{EI}W_{IE}$, which correspond to the effective weights of the disynaptic connections linking excitatory neurons through single inhibitory interneurons. (There are no direct E-to-E connections in our network). We plot the magnitude of $(W_{EI}W_{IE})_{ij}$ for each ordered pair of neurons $i \neq j$ against the overlap of the receptive fields of those same two neurons. As seen in the plot, neurons with the largest magnitude of inhibitory charge transfer tend to have relatively large receptive field overlaps; i.e., excitatory neurons that effectively inhibit each other most strongly tend to have very similar RFs. Results shown correspond to a network trained on `movie01` from the CatCam database [40, 41].

State dependence of the network selectivity as a function of the target spike rate $p_E(t_{\text{loop}})$

We also check the robustness of our results and potential history dependence of the increase in $p_E(t_{\text{loop}})$ by training the model with different initial excitatory target spike rates $p_E(0)$. In Fig 11A we plot the mean network selectivity obtained by initializing the target excitatory spike rate at $p_E(0) = 0.01$ (the baseline network used throughout the main text), 0.05, 0.07, and 0.09. We find that although the trajectories appear different, the mean selectivities are comparable at ages for which the networks have similar values of $p_E(t_{\text{loop}})$. For example, the selectivity when $p_E(t_{\text{loop}}) = 0.07$ at age 60 in the baseline network is similar to the young selectivity in the network for which $p_E(0) = 0.07$. We demonstrate this directly by plotting the mean selectivity versus p_E for each network, shown in Fig 11B. This suggests the network is approximately a state function of the excitatory target firing rate, with a weak dependence on the training history. Moreover, the results in Fig 11 suggest a lower limit of the mean selectivity of these networks, saturating at a lower bound of approximately 0.15.

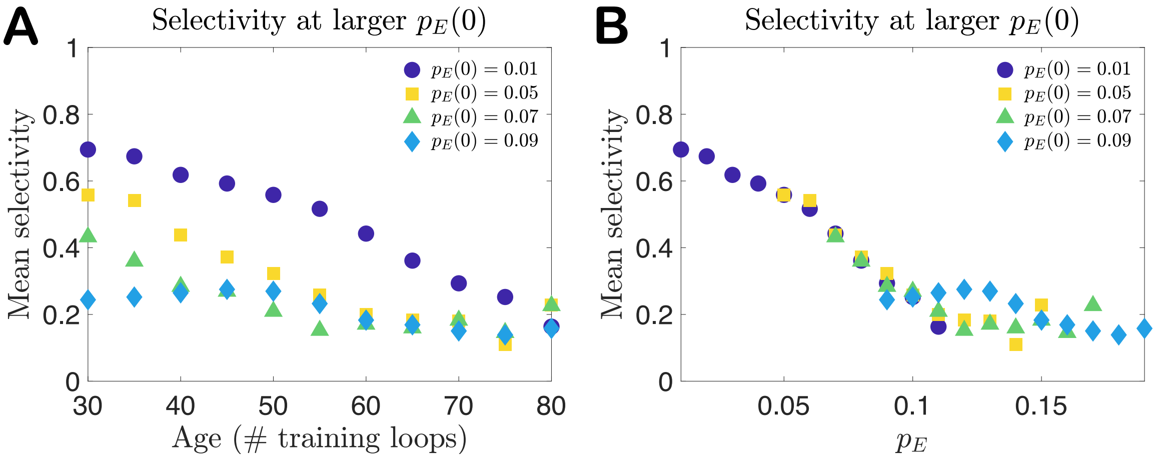

Fig 11. Mean selectivity for larger initial target firing rate: **A**. If the initial excitatory target spike rate is set to a larger value than the baseline value of $p_E(0) = 0.01$ used in the main text, the mean selectivity that develops in maturity (30 loops) is comparable to that of the baseline network when $p_E(t_{\text{loop}})$ reaches that same value. For example, the selectivity when $p_E(t_{\text{loop}}) = 0.07$ (at age 60) in the baseline network is similar to the mature selectivity in the network for which $p_E(0) = 0.07$. This appears to hold up to values around $p_E \gtrsim 0.09$, at which the mean selectivity seems to reach a lower limit of around $0.15 - 0.2$. **B**. To demonstrate this more clearly, we plot the mean selectivity versus p_E for each network (using the same data shown in **A**), showing that when the different networks have the same value of p_E they have similar mean selectivities. These results suggest the selectivity of a network is approximately a state function of $p_E(t_{\text{loop}})$. All values of p_E are measured in spikes/time-unit. Results shown correspond to networks trained on `movie01` from the CatCam database [40, 41].
