# Supplementary figures and images for: Dysregulation of excitatory neural firing replicates physiological and functional changes in aging visual cortex
Source: PLoS Comput Biol. 2021 Jan 26;17(1):e1008620. doi: 10.1371/journal.pcbi.1008620 (PMC7864437; doi:10.1371/journal.pcbi.1008620)

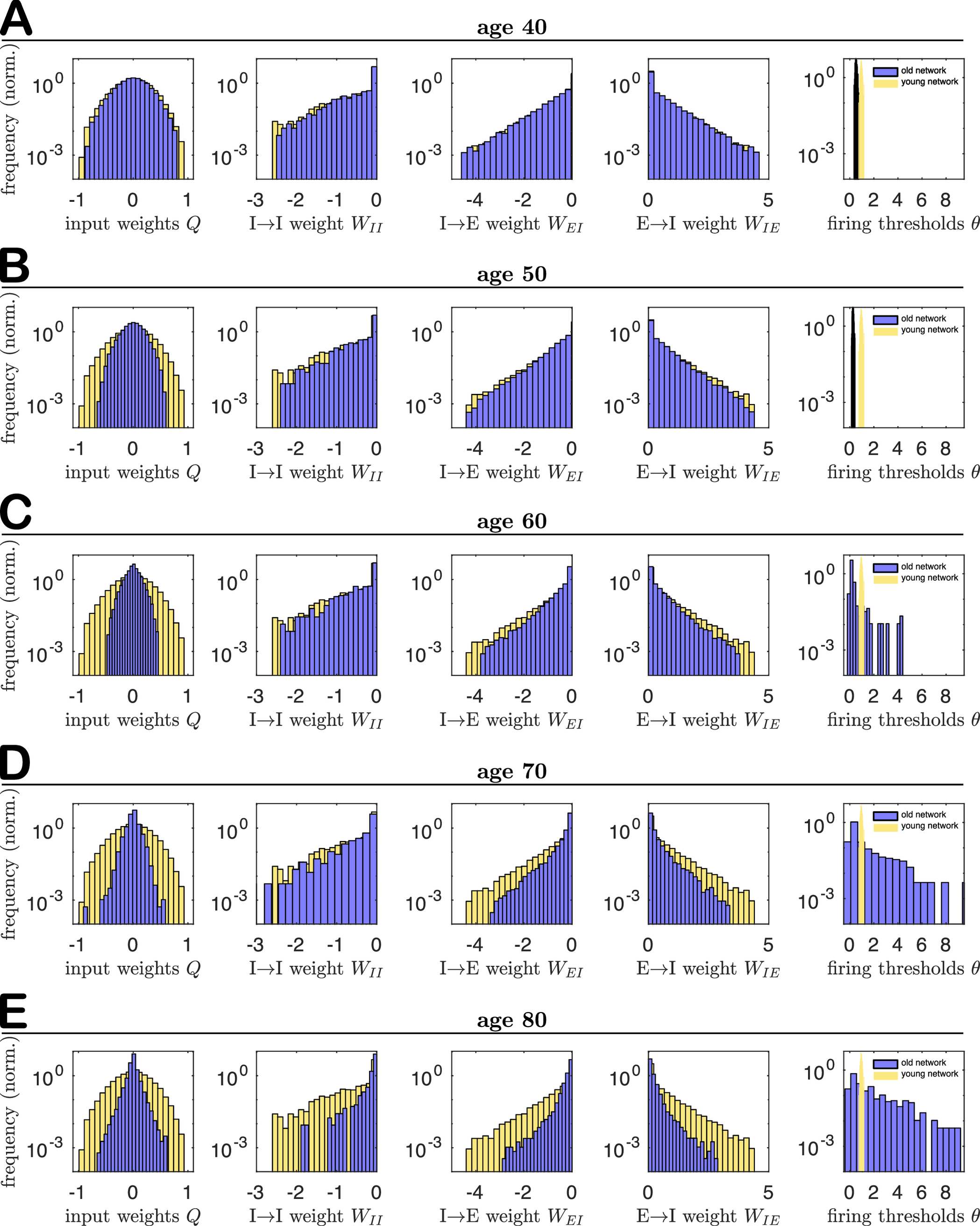

Supplement: S1 Fig — The empirical distributions of the input weights Q, lateral weights W, and firing thresholds θ at different ages during the aging process. A. 40 loops, B. 50 loops, C. 60 loops, D. 70 loops, and E. 80 loops (same data shown in Fig 2). This network was trained on frames from movie01 in the CatCam database [40, 41]. (TIF) [file pcbi.1008620.s002.tif]

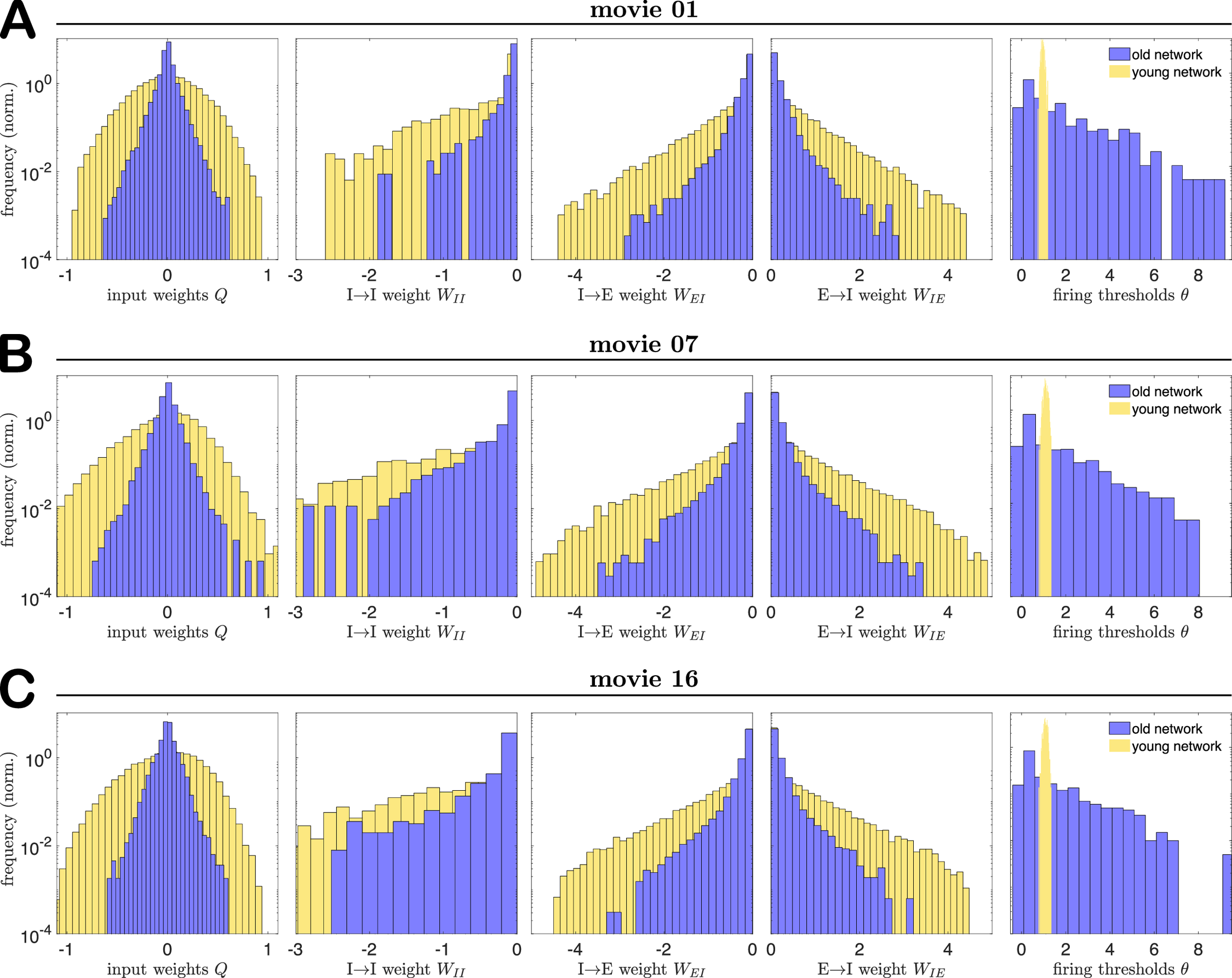

Supplement: S2 Fig — The empirical distributions of the input weights Q, lateral weights W, and firing thresholds θ obtained in networks trained on A. movie01 (same as Fig 2), B. movie07, and C. movie16 from the CatCam database [40, 41]. (TIF) [file pcbi.1008620.s003.tif]

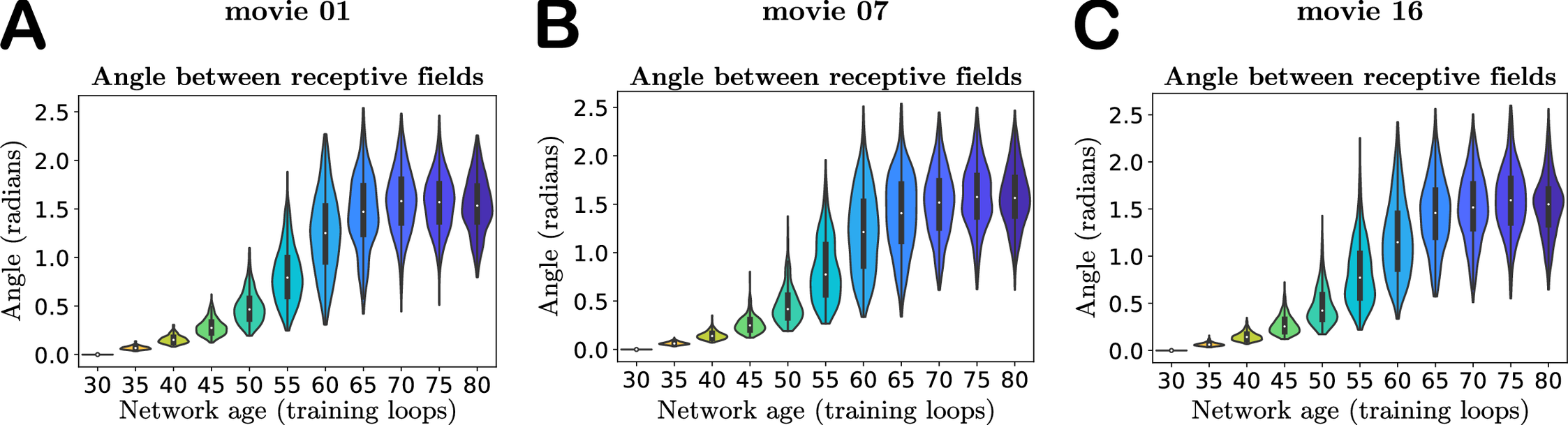

Supplement: S3 Fig — The distribution of angles between young and old receptive fields in networks trained on A. movie01 (same as Fig 3B), movie07 (panel B), and movie16 (panel C) from the CatCam database [40, 41]. (TIF) [file pcbi.1008620.s004.tif]

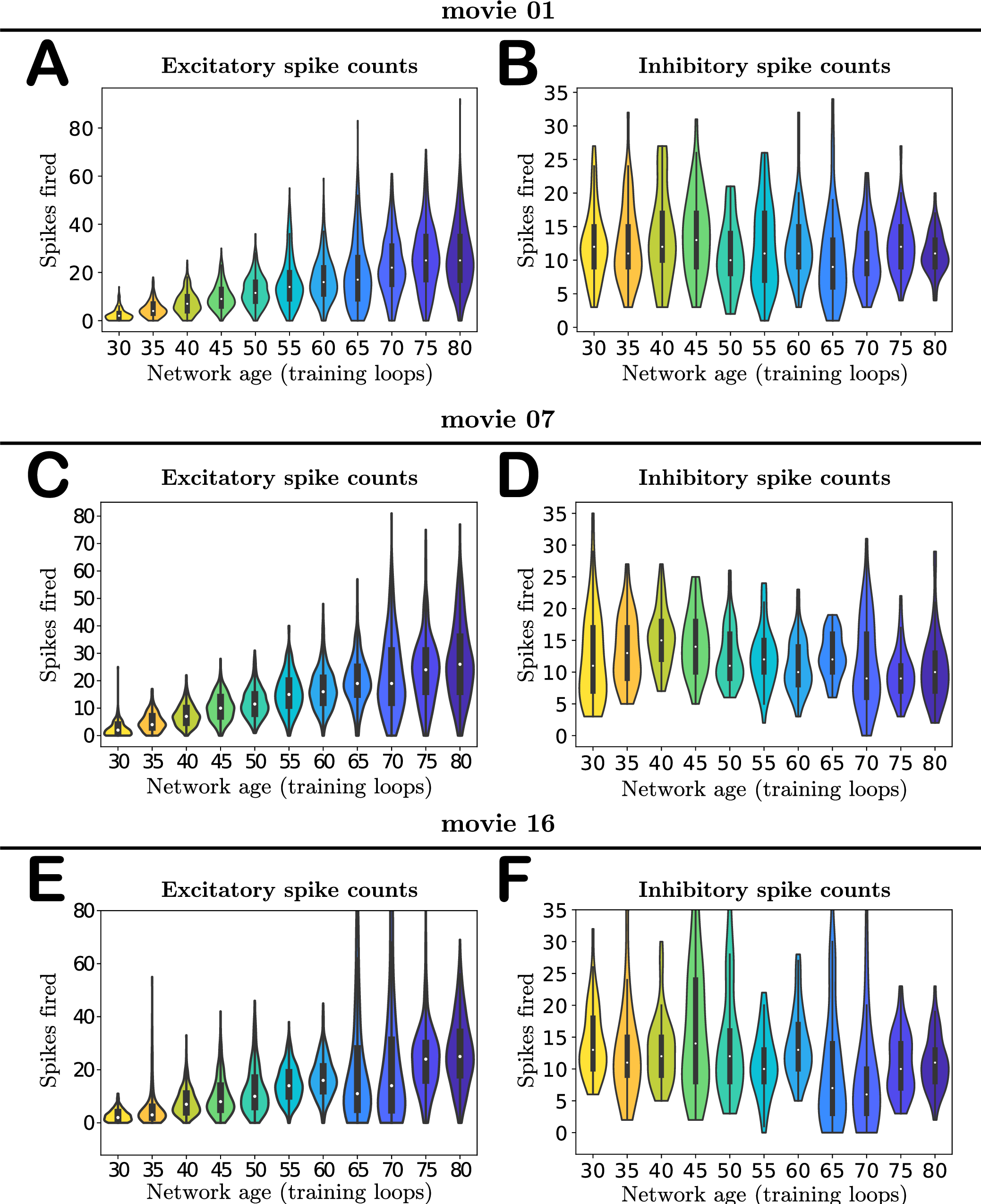

Supplement: S4 Fig — The spike counts of neurons for networks trained on movie01 (panels A and B, same as Fig 4), movie07 (panels C and D), and movie16 (panels E and F) from the CatCam database [40, 41]. In movie16 there were some outlier spike counts (e.g., the largest excitatory spike count was 243 at loop 65), so we have restricted the vertical axis to match the range observed for the other movies. (TIF) [file pcbi.1008620.s005.tif]

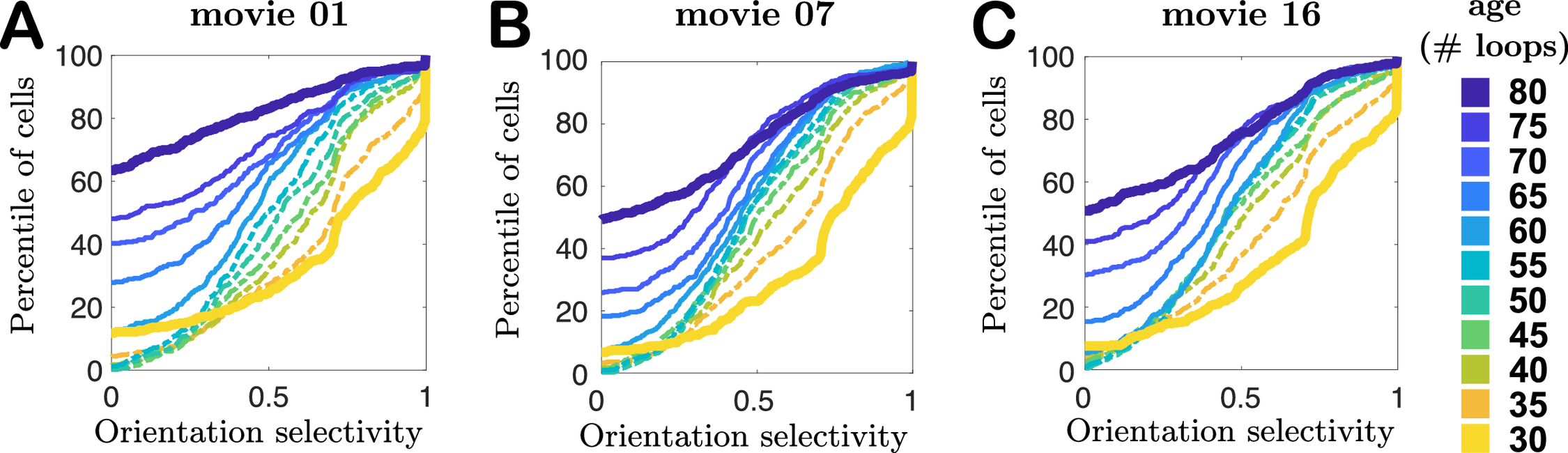

Supplement: S5 Fig — The empirical cumulative distribution functions (CDFs) for the orientation selectivity measured in networks trained on images from A. movie01 (same as panel B in Fig 5), B. movie07, and C. movie16 from the CatCam database [40, 41]. (TIF) [file pcbi.1008620.s006.tif]

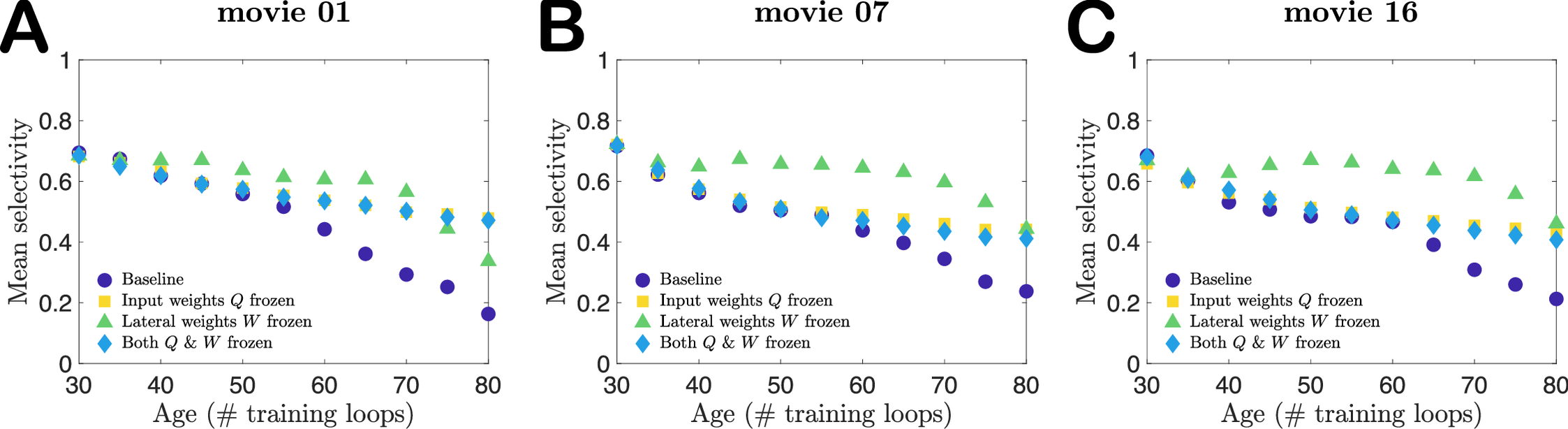

Supplement: S6 Fig — The mean orientation selectivity across neurons for networks trained on images from A. movie01 (same as Fig 6), B. movie07, and C. movie16 from the CatCam database [40, 41]. (TIF) [file pcbi.1008620.s007.tif]

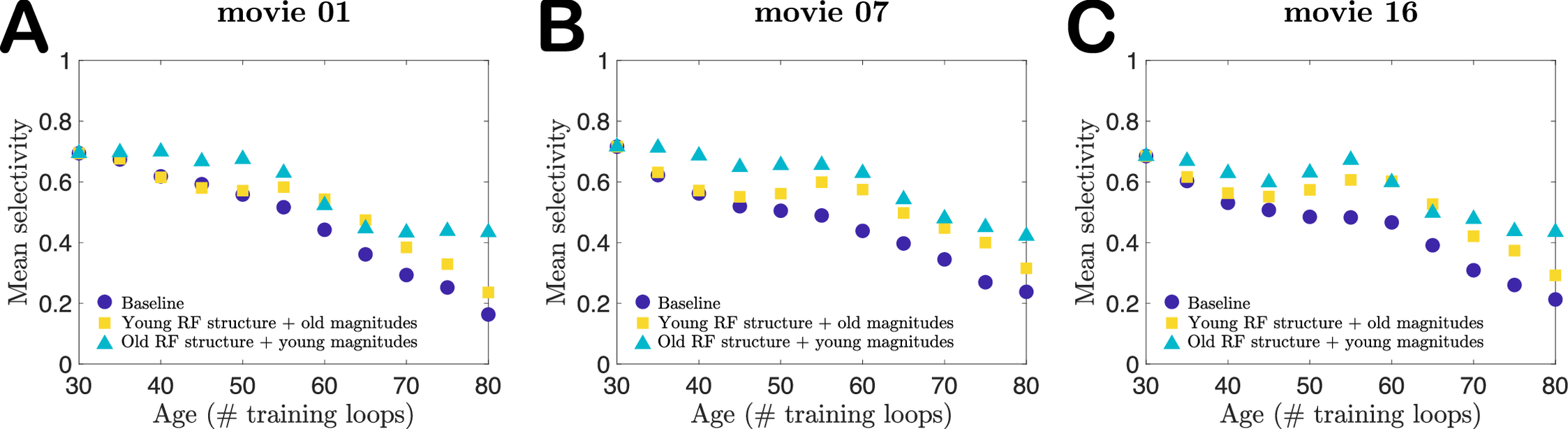

Supplement: S7 Fig — The mean orientation selectivity across neurons for networks trained on images from A. movie01 (same as Fig 7), B. movie07, and C. movie16 from the CatCam database [40, 41]. (TIF) [file pcbi.1008620.s008.tif]
